# Pseudo-puberté précoce sur syndrome de McCune-Albright: à propos d'un cas

**DOI:** 10.11604/pamj.2025.51.20.44747

**Published:** 2025-05-26

**Authors:** Kaoutar Rifai, Kaoutar El Moatamid, Hinde Iraqi, Dounia Talbi, Mohamed El Hassan Gharbi

**Affiliations:** 1 Service d'Endocrinologie et Maladies Métaboliques, Centre Hospitalo-Universitaire Ibn Sina, Rabat, Maroc,; 2 Faculté de Médecine et de Pharmacie, Université Mohammed V Souissi, Rabat, Maroc

**Keywords:** Puberté précoce, syndrome de McCune-Albright, cas clinique, Precocious puberty, McCune-Albright syndrome, case report

## Abstract

Le syndrome de McCune-Albright est une maladie rare dont le diagnostic positif est clinique et doit être évoqué devant la triade clinique: les tâches café au lait; la dysplasie fibreuse osseuse; et une endocrinopathie. La prise en charge thérapeutique dépend de l'atteinte endocrinienne ainsi que de la sévérité de l'atteinte osseuse. Nous rapportons un cas original d'une fillette âgée de 2 ans et 8 mois présentant une pseudo-puberté précoce révélant un syndrome de McCune-Albright. Le diagnostic a été évoqué devant la survenue d'une prémature ménarche et d'une prémature thélarche associées à des taches café au lait. Biologiquement, le taux d'œstradiol était élevé à 179ng/ml avec des taux de FSH, LH bas. Un test au LHRH a été réalisé objectivant un profil plat de FSH LH avec un pic LH/FSH< 1 confirmant l'origine périphérique de la puberté précoce. La radiographie du poignet a montré un aspect hétérogène de la trame osseuse en faveur de la dysplasie fibreuse osseuse. Pour freiner les saignements vaginaux et pour réduire l'hyperoestrogénie; un traitement par les inhibiteurs de l'aromatase de 3^e^ génération “Létrozole” a été prescrit chez notre patiente avec une bonne évolution clinicobiologique. Notre cas clinique illustre la nécessité de réaliser un examen clinique minutieux y compris l'examen cutanéomuqueux devant toute puberté précoce afin d'assurer un diagnostic étiologique précis et un traitement adapté.

## Abstract (en)

McCune-Albright Syndrome is a rare condition with a clinical diagnosis, typically suspected when the classic triad is present: café-au-lait spots, fibrous dysplasia of bone and endocrinopathy. Therapeutic management depends on the endocrine involvement and the severity of bone disease. We here report an original case of a 2-year and 8-month-old girl presenting with precocious puberty revealing McCune-Albright syndrome. The diagnosis was suspected due to premature menarche and premature thelarche associated with café-au-lait spots. Biochemically, estradiol levels were elevated at 179 ng/ml with low FSH and LH levels. ALHRH test demonstrated a flat FSH LH profile with a peak LH/FSH ratio < 1, confirming the peripheral origin of precocious puberty. X-ray of the wrist showed a heterogeneous bone texture suggestive of fibrous dysplasia. To control vaginal bleeding and reduce hyperestrogenism, treatment with third-generation aromatase inhibitors “Letrozole” was prescribed with good clinical and biochemical outcome. This study highlights the importance of thorough clinical examination including skin and mucosal examination in any case of precocious puberty to ensure accurate etiological diagnosis and appropriate treatment.

## Introduction

Le syndrome de McCune-Albright est une maladie génétique non transmissible qui associe des tâches cutanées hyperpigmentées, des lésions de fibrodysplasie osseuse et des manifestations endocriniennes, dont la plus fréquente est la puberté précoce périphérique [1]. Il s'agit d'une maladie rare pour laquelle les données épidémiologiques sont très limitées. Sa prévalence est estimée entre 1 pour 100 000 et 1 pour 1 000 000 à travers le monde, avec une nette prédominance féminine [2]. Le diagnostic est clinique, basé sur la triade sus-décrite. Cependant, d'autres définitions cliniques ont été apportées comme la présence d'une atteinte osseuse accompagnant toute atteinte endocrine ou cutanée caractéristique. La prise en charge globale des différentes atteintes a beaucoup évolué au fil des années [3]. Nous rapportons un cas rare et original d'une fillette âgée de 2 ans et 8 mois présentant un syndrome de McCune-Albright révélé par une pseudo-puberté précoce.

## Patient et observation

**Présentation du patient:** il s'agit d'une fillette âgée de 2 ans et 8 mois, suivie en consultation d'endocrinologie au Centre Hospitalier Universitaire Rabat pour une prémature ménarche associée à une prémature thélarche. Dans ses antécédents médicaux, la grossesse était menée à terme avec accouchement par voie basse sans incidents et sans notion de consanguinité ni de cas similaire dans la famille.

**Figure 1:**
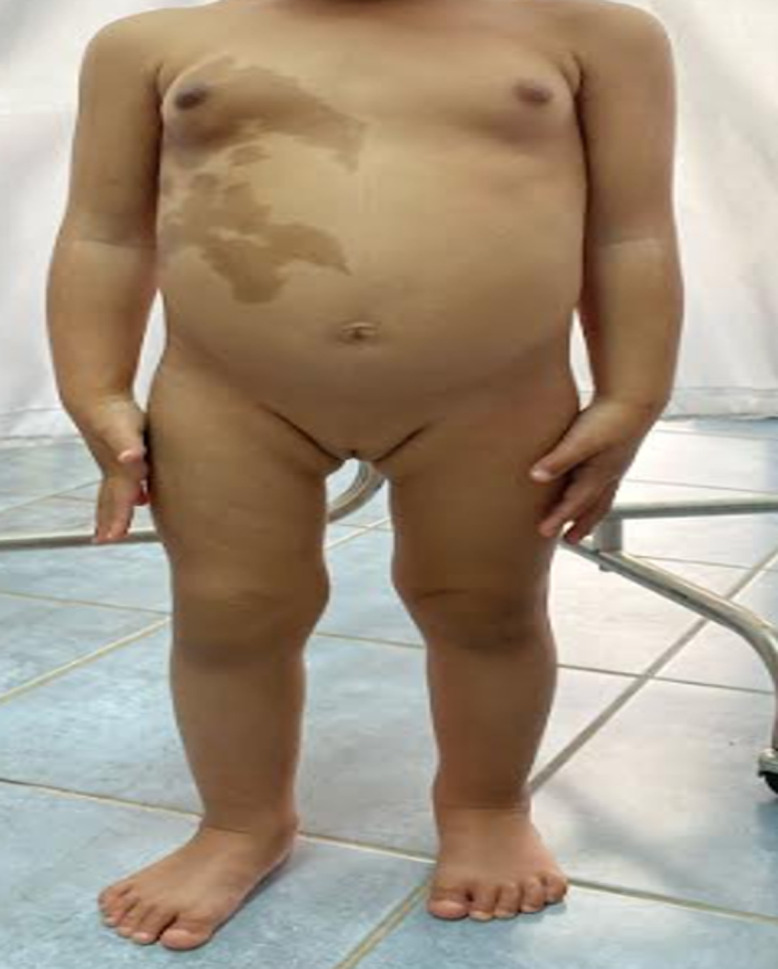
tache café au lait au niveau de l'abdomen et du thorax

**Figure 2:**
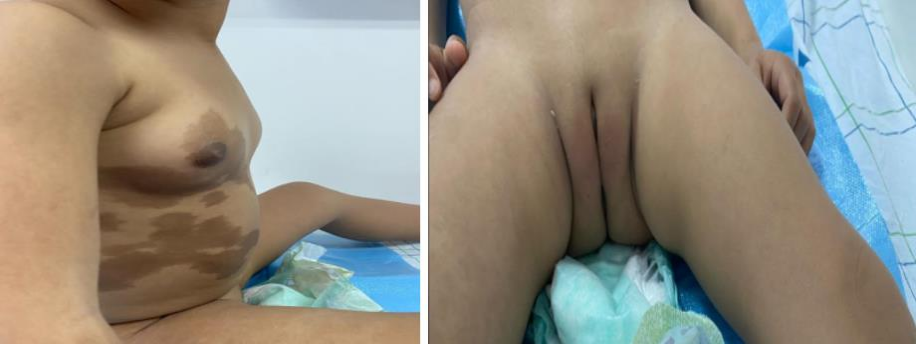
développement mammaire classé S2 et aspect des organes génitaux externes chez notre patiente

**Résultats cliniques:** l'examen clinique a objectivé une avance staturale, avec une taille à 94cm soit + 2DS, un poids à 14kg soit +1DS, associée à des tâches cafés au lait larges respectant la ligne médiane siégeant au niveau du thorax et de l'abdomen (Figure 1). L'examen des seins a retrouvé un développement mammaire classé S2 selon Tanner avec à l'examen des organes génitaux externes des grandes et petites lèvres bien individualisées (Figure 2).

**Chronologie**: le début de cette symptomatologie clinique remonte à quelques mois par l'apparition d'un développement mammaire associé à des saignements vaginaux, ce qui a motivé les parents de la patiente à consulter dans notre formation.

**Démarche diagnostique:** sur le plan clinique, le diagnostic de syndrome de McCune-Albright a été suspecté devant la présence de taches café au lait, et devant les signes de puberté précoce: prémature thélarche associée à une prémature ménarche. Devant les signes de démarrage pubertaire, un bilan étiologique a été réalisé. Le taux d'œstradiol était élevé à 179.19pg/ml avec FSH et LH basses inférieures à 0.10mui/ml. Afin de déterminer l'origine périphérique ou centrale de cette puberté précoce, un test au LH RH a été réalisé objectivant un profil plat: Les taux de LH et de FSH étaient inférieurs à 0.10mui/ml avec un rapport LH/FSH inférieur à 1 confirmant l'origine périphérique de la puberté précoce. Par ailleurs, les taux de TSH us, de FT4, de prolactine et de cortisol de 8H étaient normaux.

**Figure 3:**
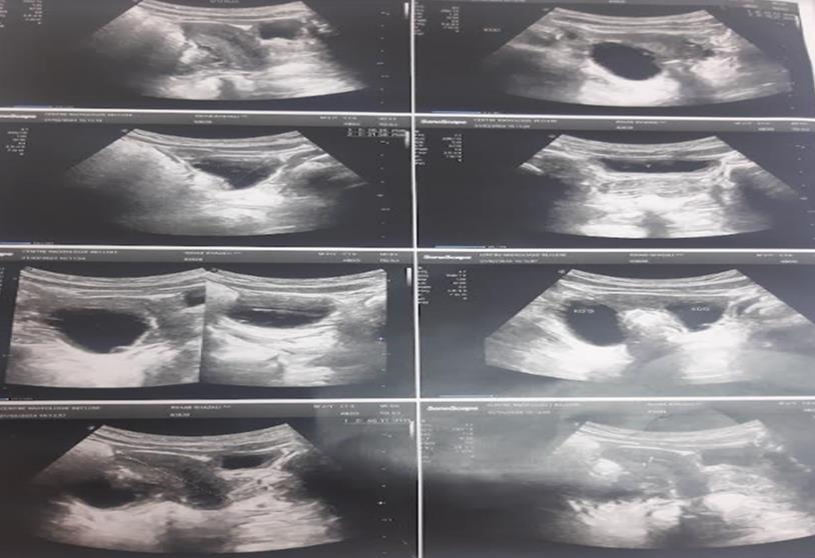
utérus augmenté de volume avec des kystes ovariens bilatéraux

**Figure 4:**
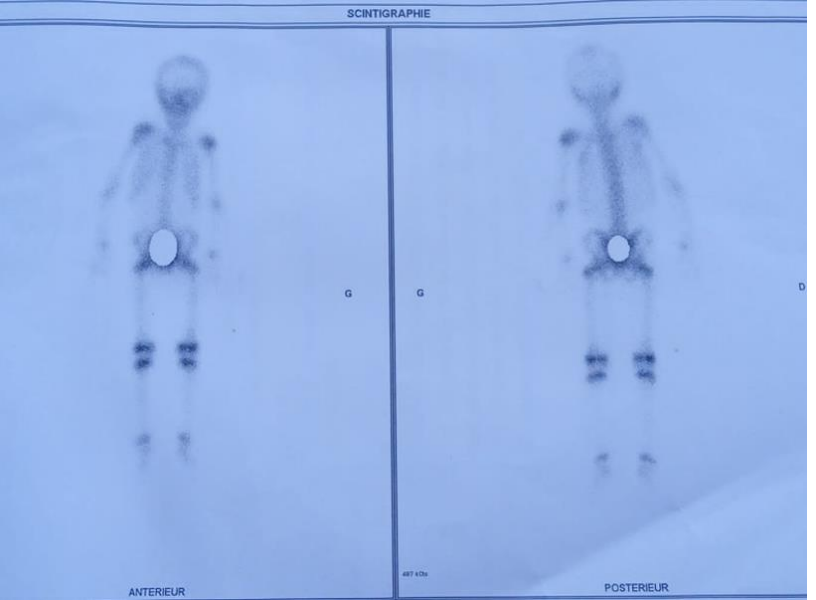
scintigraphie osseuse au technetium 99cm montrant un renforcement de fixation des épiphyses des os longs en faveur de la dysplasie fibreuse osseuse

Le bilan phosphocalcique était sans anomalie, et la patiente avait une insuffisance en vitamine D à 15.5ng/ml. Sur le plan radiologique, une échographie pelvienne a été réalisée, montrant un utérus augmenté de volume mesurant 5cm avec présence de formations kystiques ovariennes bilatérales mesurant à droite 32*20mm et à gauche 26*21mm (Figure 3). Dans le cadre de l'évaluation de la maturation osseuse, une radiographie du poignet et de la main gauche a été faite, objectivant un âge osseux avancé de 4 mois par rapport à l'âge chronologique selon l'atlas de Greulich et Pyle, avec un aspect hétérogène de la trame osseuse et un aspect vacuolé du noyau d'ossification inférieur du radius et de la jonction diaphyso-métaphysaire en faveur de la dysplasie fibreuse osseuse. Une scintigraphie osseuse a été demandée montrant un renforcement de la fixation des épiphyses des os longs correspondant à l'activation physiologique du cartilage de conjugaison, avec fixation homogène et symétrique du radio traceur sur le reste du squelette (Figure 4). En prenant en considération les arguments cliniques, biologiques et radiologiques, le diagnostic de pseudo-puberté précoce sur syndrome de McCune-Albright a été retenu.

**Intervention thérapeutique et suivi:** concernant la pseudo-puberté précoce, la fillette a été traitée par inhibiteur de l'aromatase de 3^e^ génération (Létrozole cp titré à 2,5mg) à raison d'un quart de comprimé à augmenter progressivement jusqu'à un comprimé par jour, puis réévaluation à 3 mois. La patiente a bénéficié d'une supplémentation en vitamine D et en fer.

**Suivi et résultats des interventions thérapeutiques:** après 3 mois du traitement, l'évolution clinique a été marquée par la disparition du saignement vaginal avec un aspect stationnaire du développement mammaire (S2 de Tanner). Sur le plan biologique, nous avons noté une baisse importante du taux d'œstradiol qui est devenu inférieur à 5ng/ml. Le traitement a été maintenu chez notre patiente, et le suivi se fera ensuite tous les 3 mois pour évaluation clinique et biologique afin d'évaluer l'efficacité et la tolérance du traitement par inhibiteurs de l'aromatase de 3^e^ génération.

**Perspectives du patient:** la famille de la patiente est très satisfaite de la bonne évolution clinique, vu qu'après l'introduction du traitement par inhibiteur de l'aromatase il n'y a pas eu d'autres saignements vaginaux.

**Consentement éclairé:** les parents de la patiente ont déclaré leur consentement librement et de façon éclairée, afin de permettre la réalisation et la publication de ce manuscrit.

## Discussion

Notre cas clinique illustre une cause rare de pseudo-puberté précoce sur syndrome de McCune-Albright. Très peu de cas ont été rapportés dans la littérature. Il s'agit d'une maladie rare liée à une mutation somatique faux-sens du gène GNAS sur le chromosome 20 responsable d'une activation de l'adénylate cyclase avec production excessive de l'AMPc [4]. Il a été rapporté que la plupart des patients ne présentent pas la triade classique, mais plutôt un double syndrome. Deux éléments de la triade (dysplasie fibreuse avec taches café au lait ou hyperfonctionnement endocrinien) suffisent généralement pour diagnostiquer ce syndrome, comme c'était le cas de notre patiente où le diagnostic a été posé devant l'association de tâches café au lait à la puberté précoce.

Les manifestations cliniques des patients atteints de ce syndrome varient considérablement, la manifestation clinique la plus typique étant la dysplasie fibreuse de l'os. C'est une maladie fibreuse bénigne à évolution lente, caractérisée par le remplacement du tissu osseux normal par du tissu osseux immature. Elle peut toucher n'importe quelle partie du squelette, et peut affecter un ou plusieurs os, se manifestant principalement par des douleurs osseuses locales, des gonflements, des fractures pathologiques et des déformations. Selon la littérature, jusqu'à 81% des adultes et 49% des enfants atteints de dysplasie fibreuse souffrent de douleurs osseuses, qui sont l'une des complications les plus courantes de la dysplasie fibreuse. Notre patiente ne présentait pas de douleurs osseuses cliniquement mais il y avait un aspect hétérogène de la trame osseuse faisant évoquer le diagnostic de dysplasie fibreuse.

Les tâches cutanées typiques, classiquement supérieures à 2cm de grand axe, sont de couleur café-au-lait ou marron foncé. Leurs bordures sont irrégulières et émiettées, contrairement aux tâches pigmentées observées dans la neurofibromatose de type 1, dont les limites sont très régulières. Sur le plan anatomopathologique, elles sont dues à une prolifération de macro mélanosomes. Elles se répartissent selon des localisations métamériques, et respectent la ligne médiane. Elles peuvent être présentes dès la naissance ou apparaître avec le temps [5]. Chez notre patiente, les tâches café au lait étaient présentes dès la naissance au niveau du thorax et de l'abdomen de forme irrégulière respectant la ligne médiane.

La puberté précoce atteint essentiellement les filles (30 à 50%). Elle est due à un hyperfonctionnement ovarien indépendamment de la stimulation hormonale. La présentation clinique est caractérisée par l'apparition de métrorragies très rapidement après les premiers signes pubertaires, à savoir le développement des seins et l'accélération de la croissance. On observe fréquemment la présence de kystes ovariens souvent récidivants, qui peuvent être unilatéraux ou bilatéraux. Notre patiente a consulté pour des saignements vaginaux très précoces associés à un développement mammaire en rapport avec des kystes ovariens visualisés sur l'échographie pelvienne.

Les anomalies thyroïdiennes sont la deuxième endocrinopathie la plus fréquente du syndrome de McCune-Albright (30 à 40%). Chez notre patiente, le bilan thyroïdien était normal. L'activation du GNAS peut entrainer également d'autres atteintes endocriniennes au niveau hypophysaire et au niveau surrénalien. Le traitement dépend de l'endocrinopathie et de l'étendue de la dysplasie fibreuse [6]. Un certain nombre de médicaments ont été étudiés en tant qu'approches pharmacologiques potentielles pour le traitement des filles atteintes de syndrome de McCune-Albright qui présentent des épisodes répétés de saignements vaginaux [7]. Il existe plusieurs anti-androgènes: les modulateurs de récepteurs d'œstrogènes ainsi que les inhibiteurs de l'aromatase de différentes générations dont l'efficacité a été étudiée dans le traitement de la puberté précoce du syndrome de McCune-Albright.

L'étude pilote réalisée en 2007 par Feuillan *et al*. indique que le Létrozole (inhibiteur de l'aromatase de 3^e^ génération) permet une diminution significative de la vitesse de croissance et du rapport âge osseux/âge chronologique (BA/CA), ainsi qu'un arrêt ou un ralentissement des menstruations pendant le traitement [8]. Pour notre patiente nous avons opté pour un inhibiteur de l'aromatase de 3^e^ génération (Létrozole cp titré à 2.5mg) à administrer progressivement avec une surveillance clinique et biologique tous les 3 mois de l'efficacité et de la tolérance.

## Conclusion

Bien que rare, le syndrome de McCune-Albright doit être diagnostiqué précocement afin d'améliorer le pronostic fonctionnel et de permettre un développement pubertaire normal. La prise en charge thérapeutique est multidisciplinaire associant orthopédistes et endocrinologues. En cas de puberté précoce, le traitement par inhibiteur de l'aromatase a prouvé son efficacité dans la baisse du taux d'œstradiol, le ralentissement de la maturation osseuse, et la disparition des saignements vaginaux. Des essais contrôlés et comparatifs directs seront nécessaires pour établir le meilleur traitement pour la puberté précoce dans le syndrome de McCune-Albright.
